# Dihydromyricetin induces apoptosis and inhibits proliferation in hepatocellular carcinoma cells

**DOI:** 10.3892/ol.2014.2330

**Published:** 2014-07-09

**Authors:** JIE LIU, YANG SHU, QINGYU ZHANG, BIN LIU, JUAN XIA, MINGNING QIU, HUILAI MIAO, MINGYI LI, RUNZHI ZHU

**Affiliations:** 1 Zhanjiang Key Laboratory of Hepatobiliary Diseases, Affiliated Hospital of Guangdong Medical College, Zhanjiang, Gunagdong 524001, P.R. China; 2 Department of Urology Surgery, Affiliated Hospital of Guangdong Medical College, Zhanjiang, Gunagdong 524001, P.R. China

**Keywords:** dihydromyricetin, hepatocellular carcinoma, p53, apoptosis, caspase-3

## Abstract

Hepatocellular carcinoma (HCC) is a life-threatening disease that is known to exhibit a poor prognosis. Therefore, it is important to identify an effective drug therapy for the treatment of HCC. Dihydromyricetin (DHM) is a flavonoid compound, isolated from the classical Chinese herb *Ampelopsis grossedentata*, which exhibits multiple pharmacological activities, including anticancer effects. In this study, the anticancer effect of DHM was investigated in nine different types of HCC cell lines via cell proliferation and immunoassays, as well as apoptosis detection. Two immortalized normal human liver cell lines were utilized to determine hepatotoxicity. The results revealed that DHM significantly inhibited cell proliferation and induced cell apoptosis in the HCC cell lines. However, DHM exhibited no cytotoxicity to normal human hepatic cell lines. Furthermore, it was found that DHM induced cell apoptosis in a p53-dependent manner. DHM upregulated p53 expression, and the upregulation of p53 increased the levels of the cleaved caspase-3 protein, directly inducing cell apoptosis. These results indicate that DHM is a promising candidate for the treatment of HCC.

## Introduction

Hepatocellular carcinoma (HCC) exhibits one of the highest incidences of morbidity and mortality worldwide (1), and presents the predominant histological subtype of primary liver cancer (2). As postsurgical recurrence of HCC is frequent and often fatal, surgery and liver transplant offer limited treatment options for HCC (3,4). Consequently, it is important to identify an effective drug therapy for the treatment of HCC. The anticancer ability of certain traditional Chinese medicines has been accepted in cancer therapy (4).

**Figure 1:**
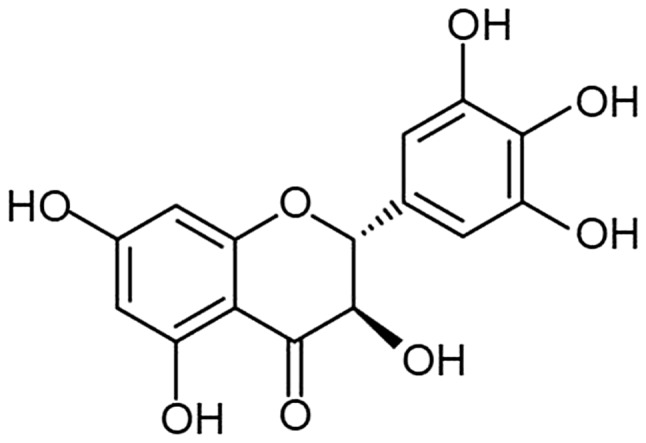
Chemical structure of dihydromyricetin.

Dihydromyricetin (DHM) is a type of flavonoid extracted from *Ampelopsis grossedentata* (Fig. 1), which exhibits pharmacodynamic effects, including scavenging of free radicals, anti-oxidative, antithrombotic and anti-inflammatory effects (5–7). In addition, DHM has been found to exhibit anti-alcoholic and anti-lipid peroxidation effects (8,9). Previous studies have suggested that DHM exhibits a protective ability on the liver. In this study, the suppression of proliferation and induction of apoptosis by DHM was investigated in hepatocellular carcinoma cell lines.

## Materials and methods

### Main reagents

DHM (Sigma-Aldrich, St. Louis, MO, USA) was dissolved in dimethylsulfoxide (DMSO) at a concentration of 50 mM, and diluted to a working concentration using culture medium, prior to use. 3-(4,5-dimethylthiazol-2-yl)-2,5-diphenyltetrazolium bromide (MTT) and propidium iodide (PI) were also purchased from Sigma-Aldrich. RNase A was purchased from Thermo Fisher Scientific (Rockford, Waltham, MA, USA). The In Situ Cell Death Detection kit, POD was purchased from Roche (Basel, Switzerland), and the fluorescein isothiocyanate (FITC)-Annexin V staining kit was purchased from BD Biosciences (Franklin Lakes, NJ, USA). Monoclonal rabbit anti-human primary antibodies against Bax, Bak, p53, caspase-3, Caspase-9 and β-actin were obtained from Cell Signaling Technology, Inc. (Boston, MA, USA). The polyclonal goat anti-rabbit secondary antibody was purchased from EarthOx Life Science (Millbrae, CA, USA).

### Cell lines and cell culture

Human hepatocellular carcinoma cell lines (HepG2, QGY7701, QGY7703, QSG7701, Huh-7, MHcc97L, MHcc97H and SK-HEP-1), one mouse hepatocellular carcinoma Hepal-6 cell line and immortalized normal human liver HL7702 and L-02 cell lines were used in this study. HepG2, QGY7701, Hepal-6 and QGY7703 cell lines were obtained from Shanghai Maternal and Child Health Hospital (Shanghai, China). L-02, MHcc97L, MHcc97H, QSG7701, Huh-7 and SK-HEP-1 cell lines were purchased from the Shanghai Cell Bank of Chinese Academy of Science (Shanghai, China). The HL7702 cell line was obtained from the Chinese Academy of Science (Kunming, China). HepG2, QSG7701, L-02, HL7702 and Hepal-6 cells were maintained in RPMI-1640 medium (Gibco-BRL, Grand Island, NY, USA) supplemented with 10% fetal bovine serum (FBS; Gibco-BRL). QGY7701, QGY7703, Huh-7, SK-HEP-1, MHcc97L and MHcc97H cells were maintained in Dulbecco’s modified Eagle’s medium (Gibco-BRL) supplemented with 10% FBS. All cell lines were cultured at 37°C in a humidified incubator with an atmosphere of 5% CO_2_.

### Cell proliferation assay

The effects of DHM treatment on the cell proliferation of hepatic cancer cell lines was detected by MTT assay. Cells were seeded in 96-well plates at a density of 1×10^4^ cells/well with 100 μl culture medium. Following 24 h, culture medium was removed and the same volume of medium, containing various concentrations of DHM, was added, respectively. Cells cultured in medium containing DMSO were used as a vehicle control. Following various periods of time, 5 mg/ml MTT solution was added to each group (20 μl/well), and then incubated at 37°C for 4 h. Next, the liquid in each well was removed and replaced with 150 μl DMSO. The absorbance was then detected using a microplate reader (PerkinElmer, Waltham, MA, USA) at a wavelength of 570 nm and the percentages of viable cells were compared with the control. The experiments were performed independently and at least in triplicate.

### Cell cycle assay

Cells were plated onto 60-mm dishes at a density of 3×10^6^cells/dish. Following overnight growth, the cells were exposed to various concentrations of DHM, and then harvested when significant proliferation inhibition was observed. Cells were fixed in 70% ethanol water at 4°C overnight, followed by incubation with 100 μg/ml PI and 100 μg/ml RNase A in PBS at 37°C for 1 h. DNA content was then determined by flow cytometry for the cell cycle distribution assay. The experiments were performed independently and at least in triplicate.

### Annexin V staining

Annexin V-FITC/PI staining was used to detect apoptosis induced by DHM according to the manufacturer’s instructions. Cells were cultured in six-well plates at a density of 1×10^5^ cells/well. Following overnight growth, cells were treated with various DHM concentrations and harvested for the apoptosis assay. Untreated cells were used as a negative control. The experiments were performed independently and in triplicate.

### Terminal deoxynucleotidyl transferase dUTP nick end labeling (TUNEL) assay

The late-stage apoptosis of hepatic cell lines was detected using the In Situ Cell Death Detection kit, POD (Roche). Cells were seeded in 96-well plates and, following DHM treatment, cells were fixed with 4% paraformaldehyde according to the manufacturer’s instructions. The cells were then counterstained with 4′,6-diamidino-2-phenylindole for 5 min at room temperature in the dark and observed under a fluorescence microscope (Olympus IX70; Olympus Corporation, Tokyo, Japan) to detect the apoptotic cells (TUNEL-positive cells).

### Western blot analysis

The expression of the apoptosis-associated proteins Bak, Bax, p53, caspase-3 and -9 were detected in hepatic cancer cell lines. Cells were suspended in lysis buffer on ice for 30 min and the cell lysates were cleared by centrifugation at 13,000 × g at 4°C for 10 min. The supernatants were then collected and detected by bicinchoninic assay. Next, the cellular lysates containing equal amounts of total protein were separated by SDS-PAGE and transferred to polyvinylidene difluoride membranes. Membranes were then blocked using 5% non-fat milk in Tris-buffered saline and Tween 20 (TBST) at room temperature for 1 h, and the membranes were incubated at 4°C overnight with the primary antibodies. The membranes were washed three times with TBST for 5 min each and incubated for 1 h with horseradish peroxidase-conjugated secondary antibodies. The bands were then analyzed by an enhanced chemiluminescence blotting detection system (FluorChem E; Proteinsimple, Santa Clara, CA, USA).

### Statistical analysis

The data were obtained from at least three independent experiments and all results are presented as the mean ± standard deviation. The differences between the groups were assessed using Student’s t-test. Comparisons were relative to untreated controls. P<0.05, P<0.01 and P<0.001 were considered to indicate a statistically significant difference.

## Results

### DHM inhibits the proliferation of HCC cell lines

**Figure 2:**
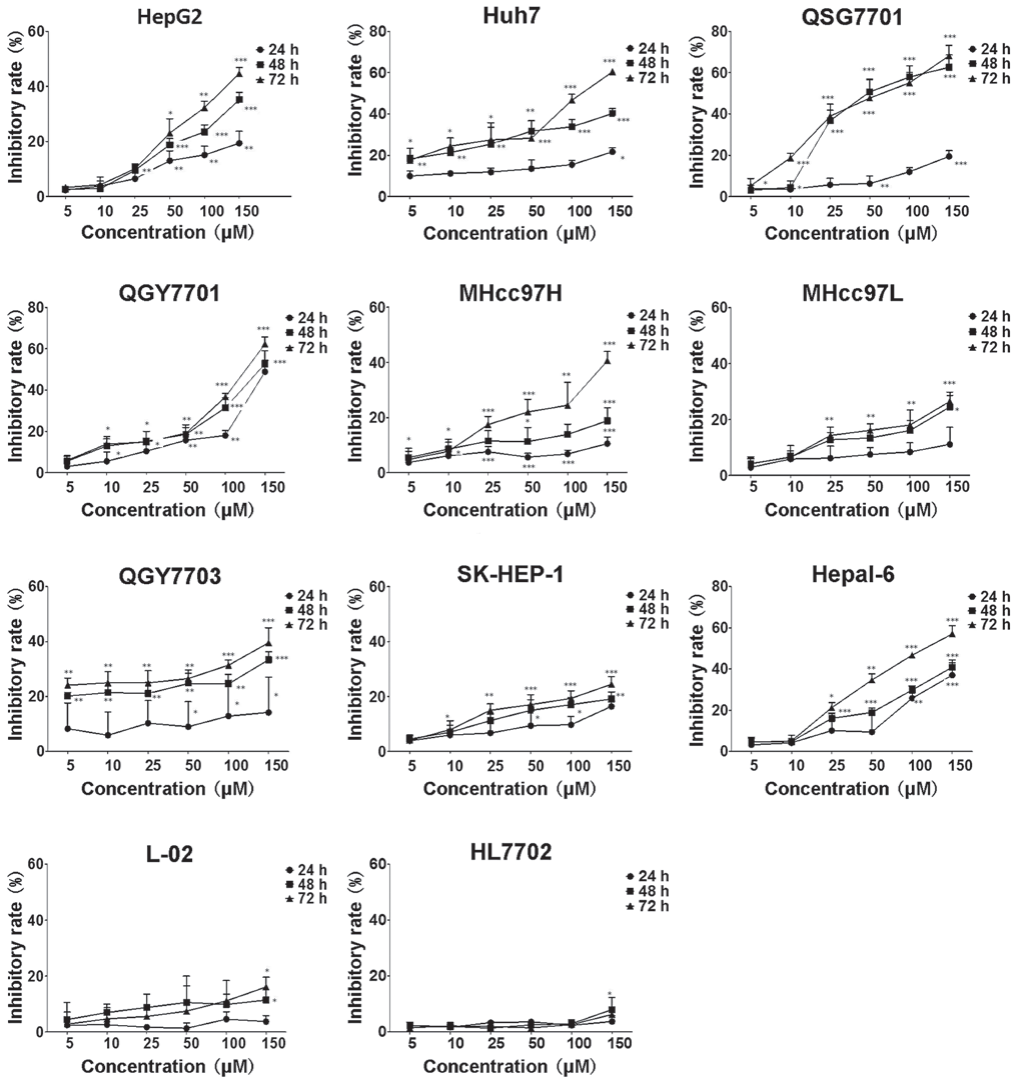
Cell growth inhibition rate of eleven different types of liver cell lines following DHM treatment. Cells were exposed to various DHM concentrations (5, 10, 25, 50, 100 and 150 μM) for 24, 48 and 72 h, and the inhibition rate of cells without DHM treatment was defined as 0. Each sample was duplicated, and the figures present three independent assays (n=4). Values are presented as the mean ± standard deviation for at least three independent experiments performed in triplicate. ^*^P<0.05, ^**^P<0.01 and ^***^P<0.001, compared with the untreated (0 μm) control. DHM, Dihydromyricetin.

MTT assay was used to investigate the potential inhibition of cell growth in HCC cells. As shown in Fig. 2, in HCC cells treated with various concentrations of DHM for 24, 48 and 72 h, cell viability was significantly inhibited in a dose- and time-dependent manner. Following 24 h of treatment with 100 μM DHM, the proliferation of HepG2, QSG7701, QGY7701 and Hepal-6 cells was significantly inhibited (P<0.01). Furthermore, following 48 h of treatment with 50 μM DHM, the proliferation of HepG2, Huh7, QSG7701, QGY7701 and Hepal-6 cells was markedly suppressed (P<0.001). The results showed that the inhibitory rate of MHcc97H, QGY7703 and SK-HEP-1 cells was significantly higher than the control group (0 μM) following 48 h of treatment with 50 μM DHM (P<0.05), while MHcc97L cells were significantly inhibited following treatment with 25 μM DHM for 72 h (P<0.01). Since the results revealed that the inhibitory rates were <50% following treatment with 150 μM DHM for 72 h, these cell lines were considered to be less sensitive to DHM administration. Notably, DHM did not affect the cell growth of immortalized normal human liver HL7702 and L-02 cell lines.

### DHM does not induce cell cycle arrest in HCC cell lines

**Figure 3:**
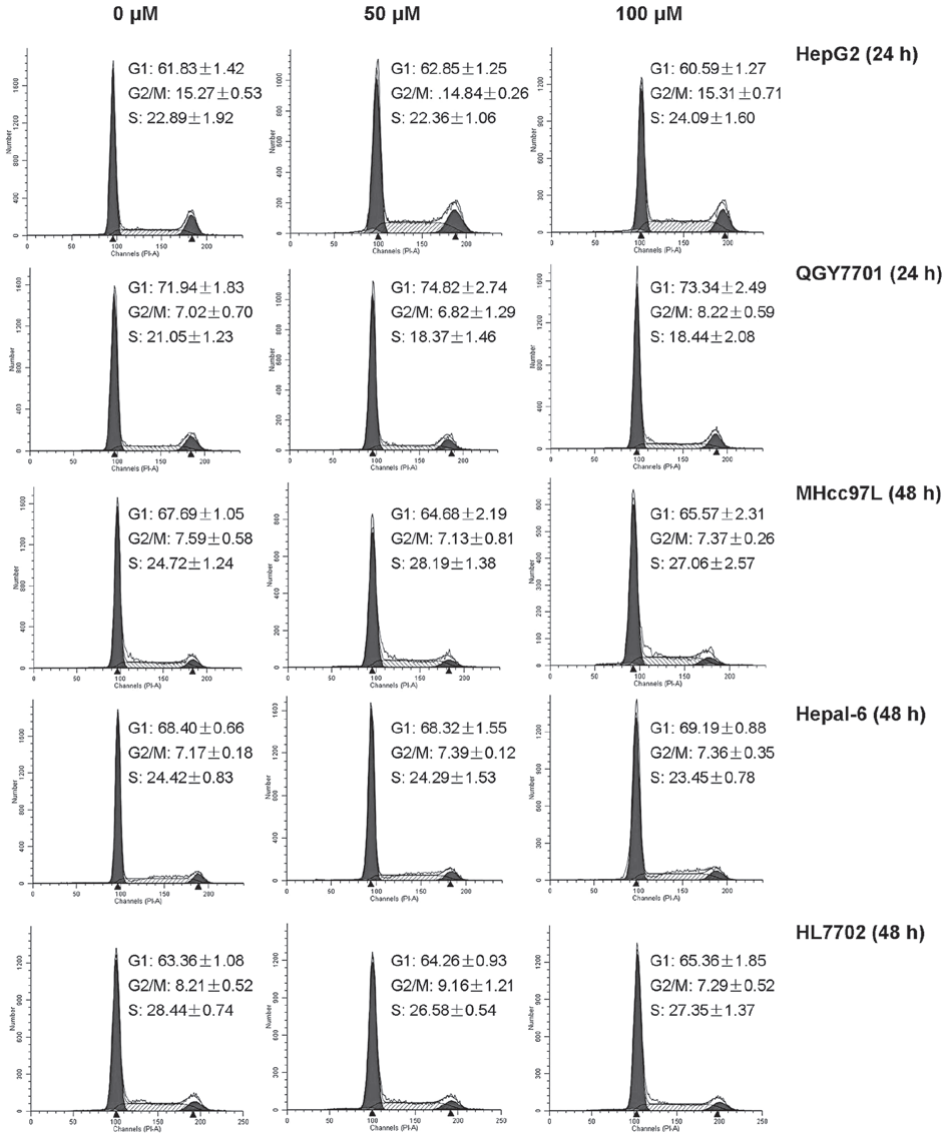
DHM does not induce cell cycle arrest. Cells were treated with various DHM concentrations (0, 50 and 100 μM) for 24 h (HepG2 and QGY7701) or 48 h (MHcc97L, Hepal-6 and HL7702) and analyzed by flow cytometry. DHM, dihydromyricetin.

The cell cycle arrest of HepG2, QGY7701, MHcc97L, Hepal-6 and HL7702 was detected by flow cytometry. No significant differences were identified between DHM-treated groups and the control (Fig. 3).

### DHM promotes apoptosis in HCC cells

**Figure 4:**
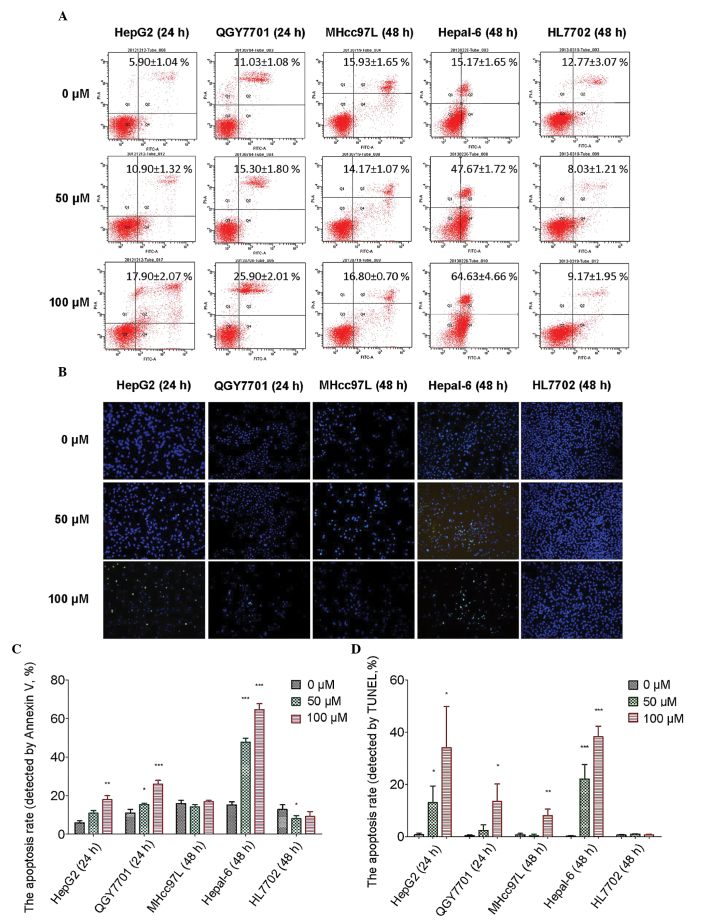
Flow cytometry revealed that DHM induces cell apoptosis. (A and B) HepG2 and QGY7701 cells were treated with various DHM concentrations (0, 50 and 100 μM) for 24 h, and MHcc97L, Hepal-6 and HL7702 cells were treated with various DHM concentrations (0, 50 and 100 μM) for 48 h, and analyzed using the Annexin V staining method. (C and D) Terminal deoxynucleotidyl transferase dUTP nick end labeling assay was used to analyze the number of late-stage apoptotic cells following DHM treatment. The experiments were independently performed in triplicate. ^*^P<0.05, ^**^P<0.01 and ^***^P<0.001, compared with the untreated (0 μm) control. DHM, dihydromyricetin.

Flow cytometry was used to detect the apoptosis of HCC cells following DHM treatment. In the present study, HepG2 and QGY7701 cells were sensitive to DHM treatment. MHcc97L cells were insensitive to DHM treatment when compared with HepG2 and QGY7701 cells. Hepal-6 is a mouse hepatoma carcinoma cell line, in which a higher apoptotic rate may be induced than in human HCC cell lines. HL7702 is an immortalized normal human hepatocyte cell line that was used as the control. Apoptosis was detected in all cell lines by Annexin V staining following treatment with DHM (0, 50 and 100 μM) for 24 and 48 h. The results revealed that following 24 h of treatment with DHM, apoptosis was induced in HepG2 and QGY7701 cell lines. DHM treatment for 48 h induced very high levels of apoptosis in Hepal-6 cells. However, apoptosis was not observed in MHcc97L and HL7702 cells following 48 h of DHM treatment. Notably, the apoptosis rate significantly decreased in the HL7702 cell line following treatment with 50 μM DHM, which indicated that lower concentrations of DHM exhibit protective effects on normal liver cells (Fig. 4A and B).

A TUNEL assay was used to detect the late-stage apoptosis of the cell lines following DHM treatment. Late-stage apoptotic cells were detected in the HepG2 and QGY7701 cell lines following treatment with DHM for 24 h, and in the remaining cell lines following treatment with DHM for 48 h. These results were consistent with the flow cytometry results (Fig. 4C and D).

### DHM induces cell apoptosis via the activation of caspase-3 and subsequent upregulation of p53, Bax and Bak

**Figure 5:**
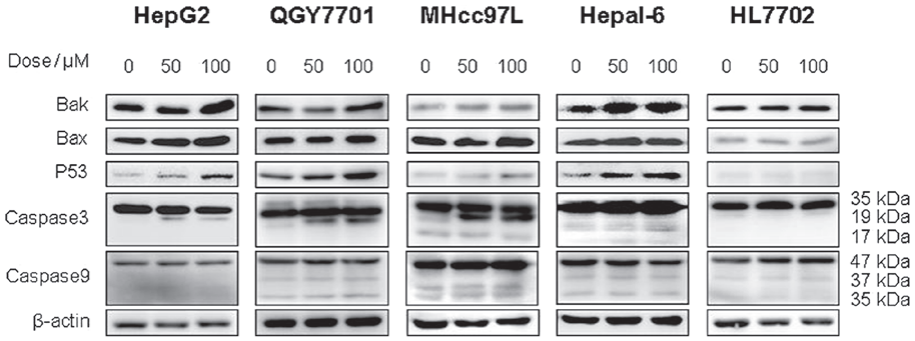
DHM induces cell apoptosis of HCC possibly via the p53/Bax and caspase-3 signaling pathways. (A) QGY7701, (B) HepG2, (C) MHcc97L, (D) Hepl-6 and (E) HL7702 cells were treated with DHM, and the levels of p53, Bax, Bak, caspase-3 and -9 proteins were detected by western blot analysis. DHM upregulated p53, then p53 recruited the activated form of caspase-3, which induced cell apoptosis of HCC in a concentration-dependent manner. No significant differences in the apoptosis-associated proteins (p53 and cleaved caspase-3) were identified following DHM treatment of the normal hepatic HL7702 cell line. DHM, dihydromyricetin; HCC, hepatocellular carcinoma.

In this study, western blot analysis was used to detect the expression of apoptosis-associated proteins in the QGY7701, HepG2, MHcc97L, Hepal-6 and HL7702 cell lines. The results demonstrated that DHM upregulates the levels of Bax, Bak, p53 and cleaved caspase-3 (p19) proteins in QGY7701, HepG2, MHcc97L and Hepal-6 cells. However, no significant differences in the expression of caspase-9 were identified. Furthermore, DHM did not affect the expression of p53, Bax, Bak or caspase-3 in HL7702 cells (Fig. 5).

## Discussion

In the present study, DHM was found to significantly inhibit the proliferation of nine different types of HCC cell line when compared with two immortalized normal human hepatic cell lines, HL7702 and L-02. The cell cycle assay demonstrated that DHM did not induce cell cycle arrest in HCC cell lines or normal human hepatic cell lines. In addition, various concentrations of DHM did not induce apoptosis in HCC cells. Notably, DHM did not induce apoptosis in the normal human liver cell lines, which suggested that DHM may present a novel candidate for the treatment of HCC based on its selective effects on liver cancer cells rather than normal liver cells.

Cell apoptosis is a complex biological process linked with intricate pathways, whereby the activation of cysteine proteases (caspases) acts as a key intracellular regulator of cell apoptosis (10,11). Specifically, caspase-3 is a key mediator in the caspase family (12). Caspase-3 may be activated by a variety of activators, which are classified into two predominant pathways: The death receptor-mediated pathway, involving caspase-8 and-10, and the mitochondrion-mediated pathway, involving caspase-9 (13,14). In the present study, no significant differences in caspase-9 expression were identified following DHM treatment, which indicated that DHM selectively induces apoptosis in HCC cells directly via the death receptor-mediated pathway.

**Figure 6:**
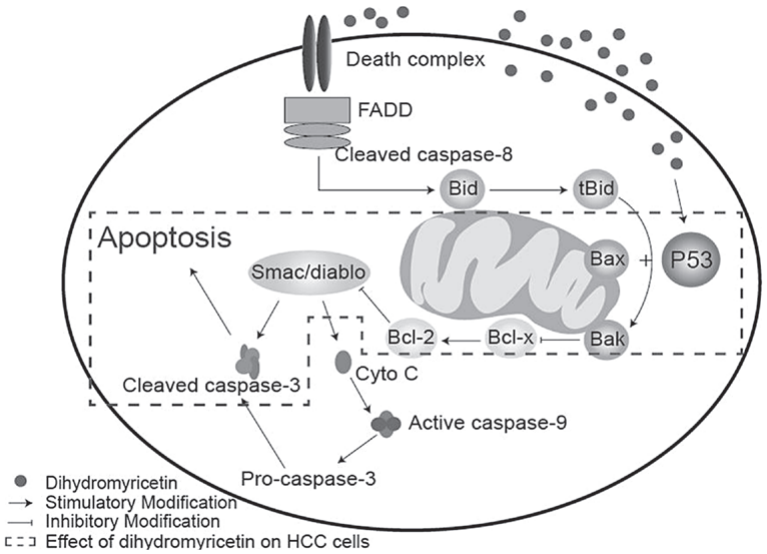
Potential mechanism of dihydromyricetin-induced cell apoptosis in HCC cells. HCC, hepatocellular carcinoma.

p53 is an important protein in the death receptor pathway, which is as an upstream regulator of pro-apoptotic Bax and Bak in mitochondria (15–17). Previous studies have demonstrated that p53 activates the transcription of Bax and Bak, whereby the activated Bax and Bak may coordinate with the release of cytochrome *c* and Smac/diablo from the mitochondria, leading to the induction of caspase-9 and -3 activation and/or cleavage, directly (17,18). In the present study, p53, Bax and Bak were significantly upregulated following DHM treatment in different HCC cell lines; however, caspase-9 was not activated. The mechanism of DHM-induced cell apoptosis may occur as follows, the upregulation of p53 positively increases the expression of Bax/Bak, whilst simultaneously inhibiting Bcl-2 protein. This then results in the activation of caspase-3, leading to cell apoptosis (Fig. 6). In addition, it was found that DHM did not affect the growth of immortalized normal human liver cells lines.

In conclusion, the results of the present study revealed that DHM effectively inhibits proliferation and induces apoptosis in HCC cells. In addition, DHM exhibited no significant hepatotoxicity to normal liver cells, which supports the possibility of DHM serving as a therapeutic candidate for HCC.
